# A Protocol for Regulating Protein Liquid–Liquid Phase Separation Using NMR-Guided Mutagenesis

**DOI:** 10.3390/mps9010026

**Published:** 2026-02-12

**Authors:** Mayu Enomoto-Kusano, Kyoko Furuita, Takashi S. Kodama, Chojiro Kojima

**Affiliations:** 1 Graduate School of Engineering Science, Yokohama National University, Tokiwadai 79-5, Hodogaya-ku, Yokohama 240-8501, Kanagawa, Japan; enomoto-mayu-zs@ynu.jp; 2 Institute for Protein Research, The University of Osaka, 3-2 Yamadaoka, Suita 565-0871, Osaka, Japan; furuita.kyoko.2f@kyoto-u.ac.jp (K.F.); tskodama@protein.osaka-u.ac.jp (T.S.K.); 3 Institute for Chemical Research, Kyoto University, Gokasho, Uji 611-0011, Kyoto, Japan

**Keywords:** liquid–liquid phase separation, nuclear magnetic resonance, NMR-guided mutagenesis, protein condensates, protein dynamics, VAPB

## Abstract

Liquid–liquid phase separation (LLPS) underlies the formation of membraneless cellular compartments, yet experimental strategies that directly connect quantitative LLPS behavior with residue-level structural information remain limited. Here, we present an integrated protocol that combines quantitative LLPS assays with nuclear magnetic resonance (NMR) spectroscopy and structure-guided mutagenesis to regulate protein phase separation. Using the VAPB MSP domain as a representative example, this workflow links residue-specific structural features to macroscopic LLPS behavior and enables suppression or enhancement of phase separation through targeted amino acid substitutions. This protocol provides a generalizable framework for systematic, residue-level regulation of protein LLPS.

## 1. Introduction

Liquid–liquid phase separation (LLPS) has emerged as a fundamental mechanism for cellular organization, enabling biomolecules to assemble into membraneless compartments such as stress granules, P-bodies, and nucleoli [1]. These condensates are formed through multivalent and weak interactions among proteins and nucleic acids and play essential roles in gene regulation, RNA metabolism, and signal transduction. LLPS is often driven by intrinsically disordered regions and low-complexity domains, making it highly sensitive to changes in sequence, charge distribution, and environmental conditions [2,3,4,5]. Despite rapid progress in LLPS research, experimental characterization of phase separation remains challenging, and careful interpretation is required to distinguish bona fide LLPS from aggregation or other concentration-driven phenomena [6]. In particular, experimental strategies that directly link quantitative LLPS behavior with residue-level structural information are still limited.

For in vitro LLPS studies, the availability of soluble and homogeneous protein samples is critical. Cold-shock expression systems such as the pCold-GST vector improve the solubility of aggregation-prone proteins and enable downstream structural analyses including NMR spectroscopy [7,8].

Optical readouts such as turbidity have long been used as empirical proxies for LLPS formation (e.g., Patel et al., 2015 [9]), but such measurements alone may not fully capture the complexity of phase behavior [10]. Recent methodological advances have enabled quantitative, high-throughput LLPS assessment using microplate-based optical measurements [11], combined with complementary microscopy [12,13].

Nuclear magnetic resonance (NMR) spectroscopy provides residue-specific insights into protein structure and dynamics and offers a rational basis for selecting residues involved in transient intermolecular interactions [13,14].

In this protocol, we present an integrated strategy for regulating protein LLPS formation by combining quantitative LLPS assays with NMR-guided mutagenesis. Using the VAPB MSP domain as a representative example, this workflow links residue-level structural information to macroscopic phase behavior, enabling systematic and reproducible regulation of LLPS.

## 2. Experimental Design

This protocol is designed to elucidate the molecular mechanism of LLPS of the VAPB MSP domain and to establish a general strategy for regulating LLPS through structure-guided point mutations. The experimental workflow integrates biochemical sample preparation, quantitative LLPS analysis, and residue-level structural characterization by NMR spectroscopy.

**Figure 1:**
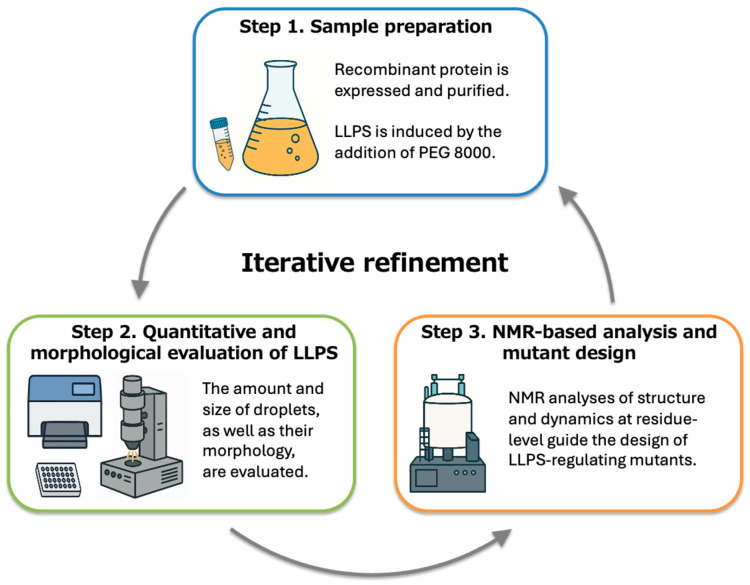
Workflow of NMR-guided regulation of protein LLPS. The schematic illustrates the overall experimental workflow described in this protocol. Following protein preparation, LLPS behavior is evaluated quantitatively and morphologically under defined conditions. Residue-level structural and dynamic information is then obtained by NMR spectroscopy under LLPS-prone conditions, which is used to guide the design of point mutants. The workflow can be iteratively repeated to refine experimental conditions and mutational design, allowing systematic regulation of protein LLPS.

As illustrated in Figure 1, the workflow consists of three iterative steps. First, recombinant VAPB MSP domain (wild type (WT) or mutant) is expressed and purified to obtain soluble and homogeneous samples. Second, LLPS formation is quantitatively evaluated by UV–Vis microplate-based light scattering and morphologically validated by optical microscopy. Third, NMR spectroscopy is employed to identify residue-specific structural and dynamic changes associated with LLPS, providing a basis for rational mutant design.

Residues exhibiting pronounced NMR spectral perturbations are selected for site-directed mutagenesis, and newly designed mutants are reintroduced into the workflow. By using these steps, residue-level structural features are systematically linked to macroscopic phase behavior, enabling rational regulation of LLPS formation.

### 2.1. Materials

1. Plasmids and Vectors: pCold-GST expression vector (3372; Takara Bio, Kusatsu, Japan) containing the VAPB MSP domain insert.
2. Bacterial Strain: *Escherichia coli* BL21 Rosetta (DE3) (70954; Novagen/Merck, Darmstadt, Germany).
3. Culture Media: Luria–Bertani (LB) broth; M9 minimal medium.
4. Affinity Resin: Glutathione Sepharose 4B (GE17-0756-01; GE Healthcare, Chicago, IL, USA).
5. Protease: Human rhinovirus (HRV) 3C protease (7360; Takara Bio).
6. Gel filtration Chromatography Column: Superdex 75 26/60 (GE28-9893-34; GE Healthcare).
7. Mutagenesis Kit: QuikChange Site-Directed Mutagenesis Kit (200518; Agilent Technologies, Santa Clara, CA, USA)
8. Chemicals: PEG 8000 (202452; Sigma-Aldrich, St. Louis, MO, USA), DTT (040-29224; Wako, Osaka, Japan), KCl (163-03545; Wako), potassium phosphate buffer reagents (164-04245, 169-04245; Wako).
9. Polystyrene 96-well microplate, flat bottom (1-6776-13; AS ONE, Osaka, Japan)
10. Silica glass beads: Spherical silica particles (43-00-701, 43-00-202, 43-00-402, 43-00-502, 43-00-802, 43-00-103, 43-00-153, 43-00-303, for 70 nm, 200 nm, 400 nm, 500 nm, 800 nm, 1000 nm, 1500 nm, 3000 nm, respectively; micromod Partikeltechnologie GmbH, Rostock, Germany)
11. Water: Ultrapure Milli-Q water (Millipore/Merck, Darmstadt, Germany)
12. Lysis buffer (Buffer A): 50 mM Tris–HCl (pH 8.0), 300 mM KCl, 0.1 mM EDTA, 1 mM DTT.
13. Elution buffer (Buffer B): Buffer A supplemented with 50 mM reduced glutathione.
14. Gel filtration buffer (Buffer C): 50 mM potassium phosphate (pH 6.8), 100 mM KCl, 1 mM DTT.
15. PEG 8000 (crowding agent): 30% (*w*/*v*) stock; final concentration 15%. Prepare one day in advance, dissolve at room temperature.
16. Potassium acetate buffer (pH 4.7): 50 mM with 100 mM KCl and 1 mM DTT.
17. D_2_O: Purity > 99.9%, 0.75 mL (31270356, Merck, Darmstadt, Germany)
18. NMR tube: 4 mmφ Symmetrical Micro Sample Tubes for Aqueous Solutions for Bruker NMR equipment (BMS-004B, Shigemi, Tokyo, Japan)

### 2.2. Equipment

- Incubator Shaker: For bacterial culture at 37 °C (BR-43FL; TAITEC, Tokyo, Japan).
- Ultrasonic Disruptor: For cell disruption during protein extraction (UD-211; TOMY Seiko, Tokyo, Japan).
- Centrifuges: High-speed refrigerated centrifuge for cell harvest and clarification; ultracentrifuge (Optima™ TLX; Beckman Coulter, Brea, CA, USA) for protein fractionation.
- Chromatography System: ÄKTA purifier for affinity and gel-filtration chromatography (Cytiva/Danaher, Washington, DC, USA).
- UV–Vis Microplate Reader: Capable of absorbance measurements from 330 to 800 nm for LLPS assays (Varioskan Flash 2.4; Thermo Fisher Scientific, Waltham, MA, USA).
- Phase-Contrast Microscope: For visualization of LLPS droplets and fusion dynamics (DMI3000B; Leica Microsystems, Wetzlar, Germany) equipped with a CCD camera (DFC series; Leica Microsystems).
- Spectrophotometer: For measuring protein concentration at 280 nm (NanoDrop One; Thermo Fisher Scientific).
- NMR Spectrometer: Bruker Avance III HD 600 MHz equipped with a cryogenic probe (Bruker, Billerica, MA, USA).

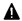

An equivalent NMR spectrometer with a frequency of 500 MHz or higher (Bruker or JEOL, Tokyo, Japan) can be used.

- Ultrasonic Cleaner: For solution mixing and degassing prior to absorbance measurements (ASU-10; AS ONE).
- pH Meter: For buffer preparation and condition control (LAQUA F-71; Horiba, Kyoto, Japan).
- Degassing Devices: Dry vacuum pump (0.095 MPa; GM-20D), vacuum filtration bell jar (3929-0001), and silicone stopper (3923-40/28) (AS ONE).
- Personal Computer: Used for NMR spectral analysis, with NMRPipe (version 9.6 or higher) [15] and Poky (BUILD 2/12/2023 or later) [16] software installed.

## 3. Procedure

The experimental workflow consists of three iterative steps: (1) sample preparation, (2) quantitative and morphological evaluation of LLPS, and (3) NMR-based structural analysis and mutant design. Repeat these steps as required to refine LLPS-regulating mutations.

### 3.1. Step 1. Sample Preparation

#### 3.1.1. Procedure 1-1. Protein Expression and Purification

- Clone the gene encoding the VAPB MSP domain into the pCold-GST expression vector.
- Generate site-directed mutants using the QuikChange mutagenesis kit (Agilent Technologies) according to the manufacturer’s instructions.
- Transform the plasmid into *Escherichia coli* BL21 Rosetta (DE3).
- Inoculate a single colony into 10 mL LB medium and incubate overnight at 37 °C with shaking.
- Inoculate the preculture into 2 L LB medium and grow cells at 37 °C until OD_600_ reaches 0.4–0.6.
- Cool the culture by shaking in an ice–water bath for approximately 5 min.
- Induce protein expression by adding IPTG to a final concentration of 1 mM.
- Incubate the culture at 15 °C for 16–24 h with shaking.
- Harvest cells by centrifugation at 3300× *g* for 15 min at 4 °C.
- Wash the cell pellet with physiological saline and centrifuge again at 3000× *g* for 20 min at 4 °C.
- Store the cell pellets at −80 °C until use.
- Thaw cell pellets on ice and resuspend them in Buffer A.
- Lyse cells by sonication on ice for 15 min (power 6.0, duty 20%).
- Clarify the lysate by ultracentrifugation (50,000 rpm, 15 min, 4 °C).
- Load the supernatant onto a glutathione Sepharose 4B column equilibrated with Buffer A.
- Elute GST-fusion proteins with Buffer B.
- Add HRV 3C protease (1 μL per 1 mg protein) and incubate at 4 °C overnight to remove the GST tag.
- Confirm tag cleavage by SDS–PAGE.
- Purify the cleaved protein by gel filtration chromatography using a Superdex 75 column (GE Healthcare) equilibrated with Buffer C.
- Collect fractions corresponding to monomeric VAPB MSP domain.
- Concentrate protein fractions using centrifugal filter units (10 kDa cutoff).
- Store purified proteins at 4 °C for short-term use.

#### 3.1.2. Procedure 1-2. Induction of LLPS

- Prepare a 30% (*w*/*v*) PEG 8000 stock solution at least one day before the assay.
- Equilibrate protein and PEG solutions at the target temperature for at least 15 min.
- Dispense purified VAPB MSP domain solution into wells of a 96-well microplate.
- Gently add PEG solution to each well without direct pipette mixing.
- Avoid introducing air bubbles during sample preparation.
- Incubate plates at 10 °C or 37 °C for 24 h to allow LLPS formation.
- For kinetic measurements, prepare samples within 10 min prior to data acquisition.

### 3.2. Step 2. Quantitative and Morphological Evaluation of LLPS

#### 3.2.1. Procedure 2-1. Microplate Reader Measurements

- Use flat-bottom 96-well polystyrene plates with a final volume of 200 μL per well.
- Measure absorbance spectra from 330 to 800 nm at 10 nm intervals.
- Control the measurement temperature between 10 °C and 37 °C as required.
- Record blank spectra (buffer + PEG) and subtract them from sample spectra.
- Normalize spectra and calculate OD_660_/OD_330_ ratios.

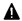

The OD_660_/OD_330_ ratio is used as an indicator of particle size. Larger values indicate larger particles.

- Perform endpoint measurements after 24 h incubation or kinetic measurements at defined time intervals.
- Measure silica glass beads (70–3000 nm) under identical conditions to obtain reference spectra.
- Fit experimental spectra to reference spectra using RMSD analysis to generate droplet size heatmaps.
- Perform at least three technical replicates for each condition.

#### 3.2.2. Procedure 2-2. Microscopic Observation

- Collect 3 μL of sample from each well immediately after microplate measurements.
- Place the sample on a glass slide and cover with a coverslip.
- Observe samples using phase-contrast microscopy at 10×–40× magnification.
- Record images and videos to monitor droplet morphology and fusion events.
- Analyze images using ImageJ (version 1.54 or higher) to quantify droplet size and circularity.

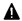

Use microscopy to distinguish LLPS droplets from aggregates; turbidity alone cannot make this distinction. Classical LLPS droplets are typically spherical and dynamic.

### 3.3. Step 3. NMR-Based Structural Analysis and Mutant Design

#### 3.3.1. Procedure 3-1. NMR Measurement

- Express ^15^N-labeled VAPB MSP domain (WT or mutants) in *E. coli* grown in M9 minimal medium supplemented with ^15^NH_4_Cl [8], and purify the protein as described in Procedure 1-1.
- Prepare purified protein at a final concentration of 0.1–0.4 mM in Buffer C (50 mM potassium phosphate, pH 6.8, 100 mM KCl, and 1 mM DTT).
- Add 150 µL of the sample containing 5% D_2_O to the outer tube of a Shigemi 4 mm NMR tube, degas the sample using a vacuum pump, and seal the tube with the inner tube, taking care to avoid the introduction of air bubbles.

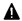

When using a Shigemi 5 mm NMR tube, add 250 µL of the sample instead of 150 µL.

- Record two-dimensional ^1^H–^15^N HSQC spectra and ^1^H–^15^N heteronuclear NOE spectra at 10 °C to evaluate the soluble state and at 37 °C to assess conditions prone to phase separation, following the procedure below.
- Set the NMR temperature to 10 °C or 37 °C.
- Insert the sample into the NMR spectrometer (Bruker).
- Control the spectrometer and perform measurements using TopSpin software (version 3.6 or higher).
- Lock the spectrometer on H_2_O/D_2_O.
- Perform probe tuning and matching.
- Optimize shim values using the TopShim module of TopSpin software.
- Determine the ^1^H 90° pulse length and power.
- For ^1^H–^15^N HSQC, load the parameter set “FHSQCGPPH”; for ^1^H–^15^N heteronuclear NOE, load “HSQCNOEF3GPSI3D”.
- Enter “getprosol 1H” (determined ^1^H 90° pulse length) (determined ^1^H 90° pulse power in dB) to set the pulse parameters corrected for the determined 90° pulse.
- Set the number of scans (NS) according to the sample concentration.
- Determine the receiver gain (RG).
- Start acquisition.
- Process the acquired spectra using NMRPipe. First, convert the raw data into the NMRPipe format. For Bruker datasets, launch the conversion GUI using the “bruker” command, which automatically generates and executes the conversion script. Next, perform Fourier transformation. In NMRDraw GUI of NMRPipe, the Macro Edit interface can be used to create, save, and execute shell scripts for spectrum processing.
- Convert the NMRPipe data into the UCSF format for Poky software using the pipe2ucsf program.
- Display each spectrum in Poky, perform peak picking, and annotate resonance assignments.

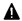

If the resonance assignments are unavailable, perform a series of NMR experiments with a [^13^C, ^15^N]-labeled sample to obtain them. If large chemical shift perturbations are observed between 10 °C and 37 °C, record additional ^1^H–^15^N HSQC spectra at intermediate temperatures and trace the signals. For the VAPB MSP domain, resonance assignments were performed at 30 °C [13]. Thus, ^1^H–^15^N HSQC spectra were recorded at 10 °C, 20 °C, and 37 °C to observe temperature dependence.

- Using the Poky “lt” command, save a list of peak positions and intensities for each residue.
- Calculate residue-specific ^1^H–^15^N HSQC chemical shift perturbations (CSPs) and peak intensity ratios at the two temperatures.
- Determine the heteronuclear NOE for each residue as the ratio of peak intensities measured with and without ^1^H saturation.
- Evaluate temperature-dependent changes in residue-specific heteronuclear NOE values.

#### 3.3.2. Procedure 3-2. Mutant Design

- Identify candidate residues for mutagenesis from those showing large temperature-dependent changes in chemical shifts, peak intensities, and/or ^1^H–^15^N heteronuclear NOE values. Prioritize residues that are solvent-exposed, likely to participate in intermolecular interactions (e.g., charged residues), and evolutionarily conserved. The CSPs primarily reflect local structural changes, while changes in peak intensities and ^1^H–^15^N heteronuclear NOE values mainly reflect alterations in the dynamics of the corresponding residues.

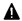

Changes in NMR features that depend on temperature can identify candidate residues that regulate LLPS. This approach applies to proteins whose condensate formation is promoted at either lower or higher temperatures. Currently, however, there is no method to unambiguously design mutants at these residues to promote or suppress LLPS formation.

- Introduce mutations by site-directed mutagenesis using the QuikChange method. Replace candidate residues with alanine residues as the initial mutation, as recommended in biochemical studies, because it often provides a straightforward interpretation of side-chain contribution. For a deeper mechanistic understanding, more refined designs are necessary (e.g., charge-conserving substitutions, such as Lys → Arg).
- Express and purify the newly designed mutants, and perform LLPS assays under the same conditions as those used for the wild-type protein.

## 4. Expected Results

This protocol is validated using the VAPB MSP domain as a representative example to demonstrate how residue-level structural information can be systematically linked to LLPS behavior through a proposed experimental workflow.

Soluble and homogeneous protein samples suitable for LLPS assays and NMR measurements are obtained for the wild-type VAPB MSP domain. Under LLPS-prone conditions, UV–Vis light scattering measurements detect reproducible droplet formation, which is confirmed by phase-contrast microscopy as spherical condensates [11,13].

**Figure 2:**
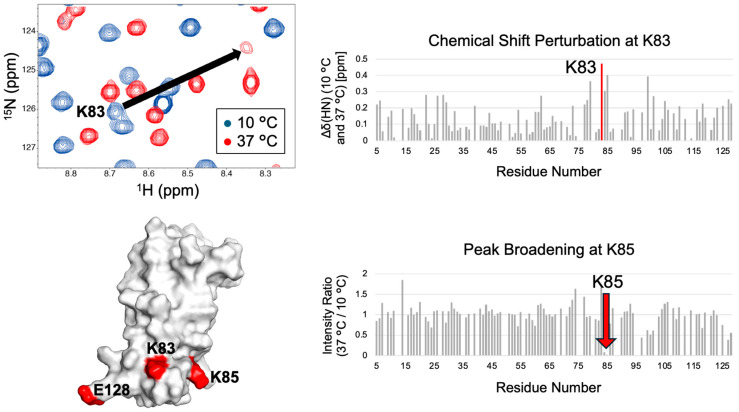
Representative expected outcomes of NMR-guided design of VAPB LLPS-regulating mutants under LLPS-prone conditions (high-temperature case). (**Top left**): Overlay of the ^1^H–^15^N HSQC spectra of the VAPB MSP domain recorded at 10 °C (blue) and 37 °C (red). (**Top right**): Chemical shift perturbations (CSPs) between the ^1^H–^15^N HSQC spectra recorded at 10 °C and 37 °C, calculated as Δδ(HN) = √{[Δδ(^1^H)]^2^ + [Δδ(^15^N)/5]^2^}. The CSP value for Lys83 is highlighted in red. (**Bottom right**): ^1^H–^15^N HSQC peak intensity ratios (37 °C/10 °C). Ratios were normalized such that the mean value equals 1. (**Bottom left**): Surface representation of the solution structure of the VAPB MSP domain (PDB ID: 9WGH). Lys83, Lys85, and Glu128 are highlighted in red.

NMR measurements performed under soluble and LLPS-prone conditions reveal residue-specific spectral changes, including chemical shift perturbations, signal broadening, and alterations in backbone dynamics. In the VAPB MSP domain, raising the temperature from 10 °C (soluble condition) to 37 °C (an LLPS-prone condition for VAPB) induces prominent residue-specific NMR changes. Lys83 shows the largest CSP, and Lys85, located close to Lys83, exhibits pronounced peak broadening (Figure 2). Residues exhibiting pronounced NMR responses at the LLPS-prone conditions, such as Lys83 and Lys85 in VAPB, are identified as candidates involved in intermolecular interactions associated with LLPS.

**Figure 3:**
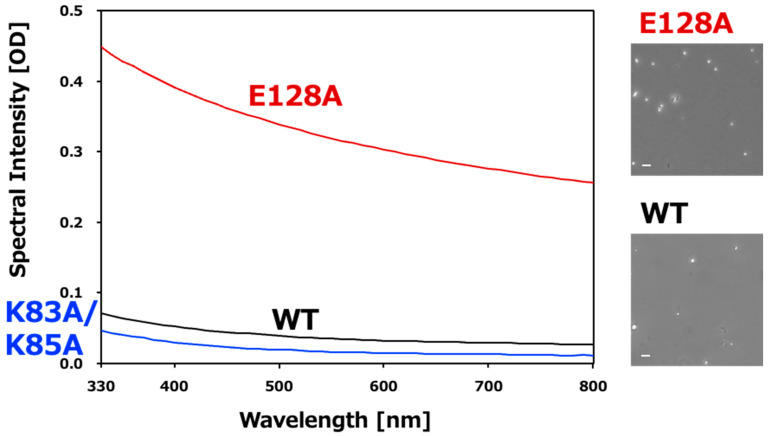
Representative expected outcomes of VAPB LLPS regulated by NMR-guided mutants. (**Left**): Turbidity measurements by UV-Vis spectroscopy [11,13]. The K83A/K85A double mutant significantly reduces LLPS formation, while the E128A mutant increases it. (**Right**): Microscopy images of VAPB LLPS confirming the formation of spherical droplets.

Guided by these observations, site-directed mutants are generated, purified, and evaluated using LLPS assays. The K83A/K85A double mutant shows reduced LLPS, whereas the E128A mutant exhibits enhanced droplet formation (Figure 3). These results demonstrate that LLPS can be suppressed or enhanced through rational mutation design.

**Figure 4:**
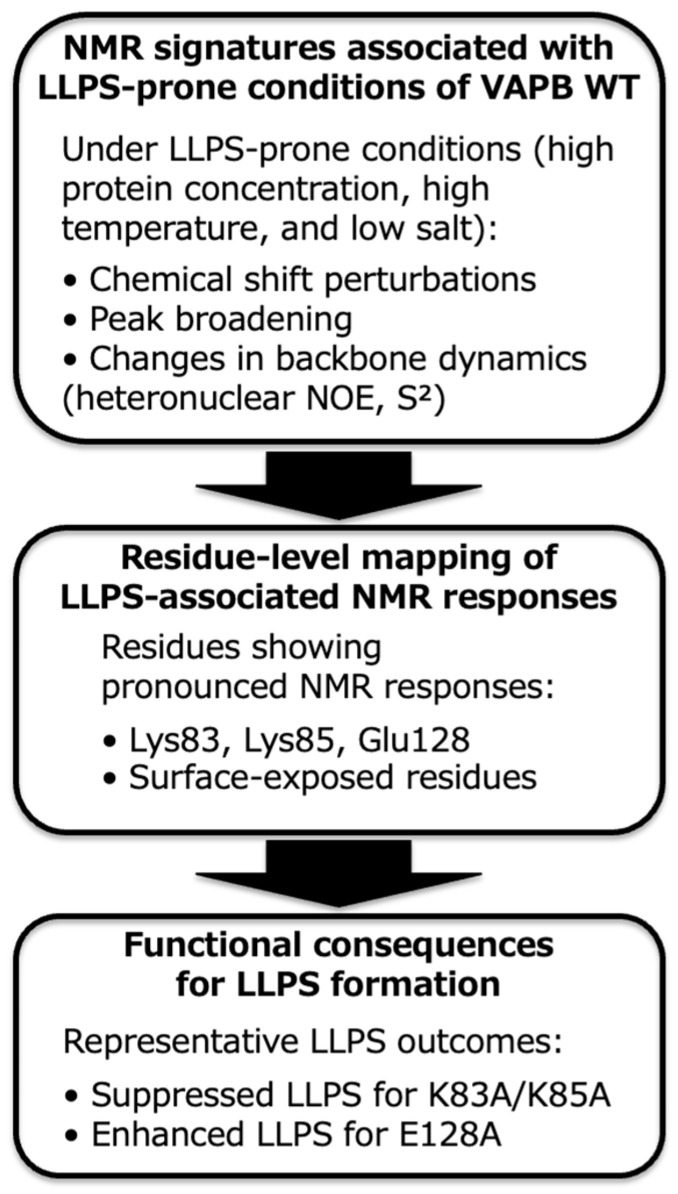
Representative expected outcomes of the NMR-guided regulation of VAPB LLPS. (**Top panel**) Under LLPS-prone conditions, NMR spectra of wild-type VAPB MSP domain typically exhibit chemical shift perturbations, peak broadening, and changes in backbone dynamics for some residues. (**Middle panel**) The residues that show NMR responses are mapped to residues that are exposed on the surface, including Lys83, Lys85, and Glu128. These residues are located close to each other. (**Bottom panel**) Mutations introduced at these positions result in different LLPS phenotypes. K83A/K85A suppresses LLPS, while E128A enhances it. This figure summarizes representative observations expected when applying the protocol to VAPB as a validation system.

By using this workflow, NMR-derived structural insights are directly translated into predictable changes in LLPS behavior. Using the VAPB MSP domain as a validation system, this protocol demonstrates that protein phase separation can be reproducibly suppressed or enhanced through targeted single or double amino acid substitutions, establishing a generalizable strategy for residue-level regulation of LLPS (Figure 4).

## Data Availability

The original data presented in the study are available upon request.

## Abbreviations

The following abbreviations are used in this manuscript:

HSQC	Heteronuclear single quantum coherence
LLPS	Liquid–liquid phase separation
MSP	Major sperm protein
NMR	Nuclear magnetic resonance
NOE	Nuclear Overhauser effect
PEG	Polyethylene glycol
S^2^	Generalized order parameter
VAPB	VAMP-associated protein B
